# Psycho‐emotional recovery, the meaning of care in the process of providing palliative care to Iranian people with cancer: A grounded theory study

**DOI:** 10.1002/nop2.1357

**Published:** 2022-09-05

**Authors:** Mir Hossein Aghaei, Zohreh Vanaki, Eesa Mohammadi

**Affiliations:** ^1^ School of Nursing and Midwifery Ardabil University of Medical Sciences Ardabil Iran; ^2^ Faculty of Medical Sciences Tarbiat Modares University Tehran Iran

**Keywords:** cancer, grounded theory, palliative care, psycho‐emotional recovery

## Abstract

**Aim:**

Despite the significance of palliative care in treating people with cancer, the provision of this type of care in Iran is vague and unorganized. This research intends to explore the meaning of care in the process of providing palliative care to Iranian people with cancer and to develop a theory that would explain the phenomenon.

**Design:**

This is a qualitative study in nature and Corbin and Strauss' Grounded Theory approach was used for data analysis.

**Methods:**

Data was collected through semi structured interviews that were held with 21 participants who have had experiences in receiving and providing palliative care. The study was conducted in April to December 2019 in palliative care centres of Tehran. Sampling first started purposefully and moved to theoretical once concepts began to emerge from the data. Comparative and continuous data analysis was undertaken using Corbin and Strauss' (Basics of qualitative research: Techniques and procedures for developing grounded theory, Sage, 2015) approach.

**Results:**

Main concerns of care providers in providing palliative care was to reduce the affliction and anxiety of patients by understanding the difficult state of patient and psycho‐emotional recovery was identified as the core category, which was performed via three critical strategies: building emotional connection, reinforcing positive mindset and having a core value in care.

## 1 INTRODUCTION

Cancer is one of the critical diseases that has caused some challenges for healthcare systems when it comes to controlling it (Agarwal & Epstein, 2018; Jacobsen et al., 2018; Romero et al., 2018) as this disease has caused many individual, family‐related and even social issues for the patients, and all of which reduce their quality of life(Abu Sharour et al., 2020; Iskandar et al., 2020; Wang et al., 2018). Therefore, taking care of people with cancer is a vital task and persistent care for these patients should be provided in a safe and supportive environment to meet their needs in an organized manner (Vinckx et al., 2018).

## 2 BACKGROUND

To achieve appropriate care for people with cancer, the development and implementation of palliative care has proved to be an effective approach and over the recent years, various countries have prioritized palliative care in their healthcare programs (Balducci & Dolan, 2016; Partridge et al., 2014; Salins et al., 2016). Analysis of current research has shown that several studies have recently concentrated on various aspects of delivering palliative care, which includes a list of the different roles that healthcare providers have in providing care, in particular nurses (Dahlin, 2015; Deitrick et al., 2011; Levit et al., 2013). Imhof et al. (2016) stated in their study that one of the most statistically significant roles of nurses is establishing and maintaining an interdisciplinary caring network, which also reflects on a nurse's leadership in the caring process (Imhof et al., 2016). To carry out this functional framework, nurses will be required to be trained and prepared, which based on the socio‐cultural context of different communities, this will differ from one place to another. Sekse et al. (2018) have also pointed, in their meta‐synthesis study, the role of nurses' availability in providing palliative care, and stated that this role may be determined based on the number of staff that a certain healthcare setting may have (Sekse et al., 2018).

Over the recent years in Iran, the palliative care approach has also gained recognition by healthcare policy‐makers; yet, the use of this approach is nascent, and only a few centres actually offer this service but they lack a purposeful programme and do not really follow a certain care model (Mojen, 2017; Rassouli & Sajjadi, 2016). Therefore, it is not clear how the care is provided and the factors that affect it or its consequences are barely considered. Rassouli and Sajjadi (2016) have shown that although some measures have been taken to develop palliative care in Iran, there has been some obstacles and since there is no transparent functional framework to carry out the care, it has been suggested that some standard functional guidelines be designed addressed to physicians and nurses to effectively perform palliative care. Khoshnazar et al. (2016) have stated that the lack of rehabilitation programmes and care instructions is a fundamental challenge in providing palliative care.

Although some studies have addressed various aspects of providing palliative care, such as spiritual care (Rahnama et al., 2014) and have explored real‐life experiences of palliative care (Seyedfatemi et al., 2014), it seems that because there is a lack of comprehensive local studies conducted on the process and methods of providing palliative care, no specific care framework has in turn been suggested. As a result, the patients who actually need these services can only receive limited palliative care since there is no clear guideline for the practice. Considering that frameworks and models can guide actions, it is necessary to develop a suitable functional model for providing palliative care in Iran. Therefore, it is necessary to develop a proper concept about the nature of palliative care and to reach a transparent understanding of the existing process of palliative care in the country. On the other hand, there are distinct and context‐specific functional areas and functions of care providers that involve institutional variations in the health system and educational differences as well.

One of the main challenges in providing palliative care in Iran is the lack of a role profile for palliative care providers and the lack of serious training in the official curriculum of medical universities (Mojen, 2017). Delivering palliative care also relies on the cultural context, patients' character and socio‐economic conditions (Stjernswärd et al., 2007). Therefore, it is hoped that this study will help to identify the factors and conditions that affect palliative care for people with cancer and the process of providing palliative care in Iran. The main purpose of this study was to explore the process of providing palliative care for people with cancer and to construct a grounded theory.

## 3 METHODS

### 3.1 Design

For this research, the qualitative Straussian Grounded Theory approach was used to explore the various methods of providing palliative care to people with cancer, to explore the current state of care in Iran, and to provide a substantive theory. Grounded Theory enables the exploration and interpretation of repeated behaviour patterns and the deduction of the underlying hypothesis to explain the under study phenomenon based on extracted data (Boeije, 2009). Since palliative care is a dynamic process consisting of a number of interactions affected by various factors (Chamberlain‐Salaun et al., 2013), Straussian Grounded Theory was considered an acceptable approach to this study.

### 3.2 Participants and data collection

The study location was the healthcare centres and charitable institutions that offered palliative care to people with cancer in Tehran. The main participants here were nurses who had experienced providing palliative care in palliative care wards and were also willing to participate in the study. Purposeful sampling was the first stage in selecting the participants. Next, participants were filtered based on the conceptual classifications that were achieved from data analysis. This allowed the collection of more accurate findings, which, in turn, allowed data saturation and the deduction of an underlying theory to define the phenomenon. Sampling and data collection continued until the saturation of all classifications were met and the underlying theory was completed. Finally, 21 individuals (9 nurses, 4 patients, 3 family members, 2 palliative care physicians, 1 psychologist, 1 spiritual counsellor, 1 social assistant) participated in this study (Table 1).

**TABLE 1:** The characteristics of participants

Participants in the purposeful sampling (number of participant)	3 Registered Nurses(RN): p1, p3, p4 1 medical‐surgical RN: p2
Participants in the theoretical sampling (number of participant)	3 RNs: p7, p10, p18 1 Psychiatric RN: P6 1 Head nurse(RN): p20 4 patients: p5, p8, p12, p16 3 family members: p9, p13, p17 2 palliative care physicians: p19, p21 1 psychologist: p11 1 spiritual counsellor: p14 1 social worker assistant: p15
Gender (number of participant)	6 males: p3, p7, p8, p14, p17, p19 15 females: other of participants
Age (years)	25–55 (mean: 42 years)
Work experience related to the subject of study (years)	3 months to 8 years (mean: 5 years)

Data collection was undertaken by means of interviews. The unstructured interviews first started with open‐ended questions such as “Please share your experience with providing palliative care to patients” and “Please share your experience in dealing with patients' problems and issues at your workplace,” in order to collect the participants' relevant experience. Then, based on their answers, participants were asked exploratory questions, which were later followed by other questions that concerned their actions or the events, the reason for selecting certain treatments and way they had offered them. This was done by asking questions like “Could you please describe exactly how you perform your tasks?” and “Could you please elaborate.” These questions made in‐depth analysis possible. Then, by analysing all data collected from purposeful interviews, supplementary interviews were performed and theoretical sampling was used to determine the features of the phenomenon. All interviews were face‐to‐face and carried out at places of participant's choosing. The interviews lasted for an average of 47 min and recorded upon the participant's consent.

### 3.3 Data analysis

Data analysis was performed in parallel with data collection and constant comparative analysis were used. The researcher was involved in the data collection process and the transcribing of the audio files. Then, the researcher read the transcriptions a number of times in order to reach a deep and general understanding of the collected data. These results influenced and guided subsequent interviews. In addition, the memoing was used to confirm data saturation, to confirm principles and to complete the underlying theory for building emotional connection, having a core value in care, reinforcing a positive mindset and tending psychological distress in care providers. Corbin and Strauss' (2015) approach was used for data processing, which consists of the following steps.

#### 3.3.1 Open coding to identify concepts

As soon as the first interview was over, open coding began. After studying the transcriptions of the interviews for several times and reaching a better understanding of the nature of the content, the text of the interview was divided and some parts were underlined. A title was selected to define and later used to refer to that certain piece of information. The title was either extracted from the participant's own words used in the interview (in vivo codes) or in some other cases, the researcher selected a title to define the concept.

#### 3.3.2 Development of concepts based on their features and dimensions

The lower level of categories was defined by comparing the data collected between interviews that were in line with the concepts and by asking questions that allowed the concepts to be further established through theoretical sampling (memo writing). The categories where connected based on their conceptual, featural and dimensional characteristics. By considering the evolved concepts, the main concerns of care providers were identified. As an example, Table 2 demonstrates how the concept of Building Emotional Connection was developed through stages one and two.

**TABLE 2:** The formation and development process of building emotional connection concept

Category	Subcategory	Main Codes	Quotation
Building emotional connection	Developing and maintaining a cordiality relationship	Building a kind and calm relation with the patients (P7. P8. P9, p18)	We communicate with our patients with calmness and kindness.
		Using kind and heart‐warming words when communicating with patients (p2. P16*2)	We use warm words like dear father or dear mother
		Building a receiving and open relation with the patient (p6. P9*3. P10*2)	I have always tried to attend a patient with a smile.
		The care providing team is open and receiving of patient's questions (p9*4. P13*2. P14*2. P15)	I always gladly answer patients' questions
	Being present beside the patient	Nurse is present at the patient's bedside (P1*6. P2. P3*3. p4*2. P7*5. P9. P10*3)	I understood that s/he wanted someone to be beside him/her, the patient took my hand and I saw that s/he needed attention so I stayed beside him/her a while longer
		The nurse is always available and present for the patient (P7. P9*3. P13)	There have been times that I have even given my mobile number to patients and told them that they can feel free to call me whenever, whether I am on a shift or not, they can always call for a consult and I will help
		The constant presence of the patient's family beside them (P4. P5. p6. p11. P9. P13*5)	His/her family was constantly beside him/her. The presence of his/her parents and brother somehow calmed the patient.
	Mutual understanding with the patient	Accepting and understanding the patient's condition (P3. P6*2. P10. P13. p14)	In that situation what I could do was to accept the patient's condition as it was and to understand him/her
		Encouraging the patient to express their emotions (P1. P5. P11*2. P14)	I told him/her that I understood s/he is in pain and has many problems, and that s/he is afraid of what might happen in the future. As soon as I said that s/he opened his/her heart to me.
		Putting oneself in patient's shoes (p11, p20)	I understand how they feel, I try to put myself in their shoes and view the issue from their perspective

#### 3.3.3 Analyse data for context

Here, based on data analysis, the researchers tried to reveal the situations that had affected the participants and caused them to respond in a certain way.

#### 3.3.4 Analyse data for process

To search for the process, the researchers read the notes of previous analyses. By identifying the context and condition of the palliative care process, the researchers sought to identify the strategies and behaviours that participants exhibit in response to problems resulting from the impact of contextual conditions. Therefore, by continuously analysis and comparing categories and subcategories, and by evaluating the memos and processes inferred from the interviews, an effort was made to obtain more abstract categories and to clarify the strategies used by the participants in dealing with the context.

#### 3.3.5 Integrating classifications

After carefully reviewing and categorizing all the memos, recording the narrations, and drawing a diagram, the researchers looked for cues that reflected on how categories were arranged, and eventually marked the core category.

### 3.4 Rigour

In order to ensure the validity and accuracy of the study, the Lincoln and Guba's criteria of credibility, dependability, confirmability and transferability have been used (Polit & Beck, 2008). Based on the strategy of long‐term interaction with data (which in fact increases the validity and accuracy of the data) the researcher spent as much time as required to collect enough data that would allow a deep understanding of the participants and ensure class saturation. Also, the member‐checking technique was used for enhancing the credibility of the data and the findings through which we summarized and reviewed each interview content once holding it and asked the corresponding interviewee to confirm or revise our perceptions. Besides, a copy of each generated codes was provided to the corresponding interviewee and he/she was asked to revise or comment on our analyses. Accordingly, the findings were revised based on interviewees' comments.

The auditing technique was used to ensure the dependability of the findings. Accordingly, all the phases and the trends of the study were meticulously recorded and reported in order to provide others with the opportunity to trace our research‐related activities.

To guarantee confirmability, we explained in detail all phases of the study including data collection, data analysis, conceptualization and categorization. This activity helps external reviewers assess and scrutinize our activities.

To increase transferability, attempts were made to enable others' judgement and evaluation of the transferability of the data through providing detailed descriptions. Additionally, applying maximum diversity in sampling, including gender, age and work experience, enhanced the transferability of the findings. Besides, comparing the findings of this study with the findings of previous studies helped enhance the transferability of the findings.

### 3.5 Ethical considerations

First permission was obtained from the ethics committee of the Tarbiat Modares University to carry out this study. Then, written consent forms were designed and given to participants to fill in and it was received. Prior to giving out the forms, the purpose of using a voice recorder during the interviews was explained to them and upon their consent the interview was taken. The time and place of the interviews were determined with the participants' consent and based on their choosing. Participants were assured that their statements were kept confidential; so that their data has been managed, analysed, and reported anonymously, using numbers. In addition; participants assured that they would be free to exit the study at any stage.

## 4 FINDINGS

By continuously comparing data through analysis, the following main categories were found: Building emotional connection, reinforcing patient's positive mindset, having a core value in care, understanding the difficult state of patient, cooperation of various care resources, inner peace of patients and psychological distress of healthcare providers. Healthcare providers have tried to reduce the affection and anxiety of patients by understanding their difficult state; and to this end, they have applied different strategies that have constructed a care process that is explained in detail below.

### 4.1 Reducing affliction and anxiety in patients (main concern)

The main concern of the study participants in terms of palliative care delivery was reducing their affiliation and anxiety. People with cancer normally encounter devastating conditions, which is the result of the mental, social, spiritual and physical turmoil that they experience. Care providers seek to reduce the agitations that have developed in the patient's mind to ease their condition. A Psychiatric Registered Nurse explained a relevant experience in the interview:

When these patients undergo chemotherapy, when they wake up, they check their pillow to see how much hair they have lost, I mean, it's like they have drowned themselves in such an atmosphere. I spend time with them and explain the process so that their mind could be relieved from all the anxiety they're experiencing (p 6).

In the theoretical sampling, a psychologist mentioned how essential it was to reduce frustration and control all the emotions that may lead to depression in patients:

We try to control patients' frustration to the point where they won't pull back anymore. This frustration is not something simple, there is a lot going on in the background, we try to understand the background that has built up to it. We try to reduce the emotions and affliction that cause frustration in them (p 11).

### 4.2 Understanding the difficult state of patient (context analysis)

Analysis of data shows that care providers understanding of the challenging circumstances of patients with cancer, that means in terms of observing and understanding the lengthy tedious nature of their disease and treatment, patients drown in negative emotions, the patient's non‐compliance because of their condition and patient's vague future and opporunity of death are formed. When care providers take underlying factors into consideration, they try to reduce the patients' affliction and anxiety in order to overcome this main concern. For instance, a Registered Nurse shared the following about observing and understanding the lengthy and tedious nature of the patient's disease:

They are tired of the treatment process and their long‐term hospitalization, from being diagnosed with the disease until they are cured. It's the nature of their disease and since they are hospitalized here every time, it has become tedious for them. They have this destructed state … we understand this condition of patients and we try to reduce their suffering (p 3).

Psychiatric RN shares the following about understanding how patients may drown in their negative emotions:

Some conditions are really hard for patients and they drown themselves in these atmospheres… When I see them in these conditions, I try to treat them in such way that would reduce their affliction and anxiety so at least they won't drown in all the emotional negativity (p 6).

### 4.3 Analysing the process

Data analysis showed that the main strategies used by palliative care providers in treating people with cancer to reduce their affliction and anxiety, were recovering patients by building an emotional connection with them, having a core value in care providing, and reinforcing a positive mindset in them. These strategies induce inner peace in patients and they cause psychological distress in care providers.

### 4.4 Building emotional connection

An emotional connection includes features and dimensions that are being present beside the patient, establishing a cordiality relationship with them, and finally, reaching a mutual understanding about them and their condition. A Registered Nurse shares a relevant experience:

During a shift, I was by a patient's bedside when she held my hand and said that her heart was racing. I understood that she wanted someone to be beside her to talk to, so I stayed little longer and spent some time with her (p 1).

A spiritual counsellor shared the following about developing and maintaining a close relationship with patients:

We visit patients daily and ask them to tell us how they feel. Well, these patients are somehow experiencing a chaos, so we try to develop and maintain a close relationship with them (p 14).

A psychologist shared the following about reaching a mutual understanding about the patient and his/her condition:

We allow them to fully understand their condition, we let them pour their hearts out and feel safe with us, and when this happens, we try to tell them that we understand what they're going through (p 11).

### 4.5 Having a core value in care

The having a core value in care strategy is the focus of the provided care, which is expected to be whatever the patient values. Here, care providers respect the patient's existential beliefs, try to meet the patient's spiritual needs as far as possible and provide caring services with dignity—all of which are efforts to reduce the patient's affliction. A Registered Nurse shares the following about respecting patients’ beliefs:

We don't try to impose our own beliefs on patients. We let them talk and we just point them into a certain direction; for example we would ask have you ever thought about death? How much do you think about it? What do you think about? How do you feel about it? (p7).

About paying attention to the spiritual needs of patients in order to reduce their affliction and anxiety, another participant shared:

We had a patient who felt hopeless and empty, and I had given the Quran to him many times and would tell him to recite one or two verses of it when he felt like that. In some way, I tried to build a spiritual atmosphere to help the patient overcome the empty feeling that he was experiencing (p 2).

About dignity‐based care, care providers have tried to respect patients' privacy and decisions and have tried to consider each patient as a unique case. A social worker shared the following:

The patient him/herself is important to us, and the way s/he thinks or feels. This is important to us as it determines how we get to address the patient's problem (p 15).

### 4.6 Reinforcing patient's positive mindset

Data analysis showed that the affliction and anxiety, which patients' experience, makes their treatment a tedious process for them, which eventually may lead to mental and psychological distress. In this regard, care providers have tried to use a positive mental reinforcement strategy where they try to adopt methods that raise the patient's spirit, induce real hope and instil reality in the patient and increase care awareness in the patient. The palliative care physician claimed the following about improving the patient's spirit to relieve them from emotional mental stagnation:

These patients, they see themselves as if they are in hell and undergoing all the pain and torment in the world and can't get out of it. So, anyways, I just want to raise their spirit. Give them some energy to reduce the anxiety and chaos that they are going through, and help them to get out of stagnation (p 21).

About the effect for inducing real hope and instilling reality in patients, a Registered Nurse shared the following:

You can't give these patients false hope, so we don't lie to patients or give them false hope, we don't say that don't think like that you'll get better. We try to control this hopelessness so that the patient doesn't commit suicide or something and believe that there is no hope for them (p 3).

Also, another RN shared the following about the relation between care awareness in patients:

Here, we tell the patient beforehand about what is going to happen. It's important that they have complete awareness about what they should be expecting so that they can cope better with the side‐effects and pain later … (p 4).

Data analysis showed that factors affect the use of these three strategies by care providers. Lack of sufficient human resources and high workload of care providers disrupt these strategies, while factors such as the homogeneity of demographic and cultural characteristics and the cooperation of various care resources facilitate their use.

The high workload and insufficient human resources to provide care have reduced the significance of building an emotional connection with the patient because in such circumstances the nurses do not have that much time to give to patients anymore. The head nurse of palliative care ward explained:

There are a great number of patients hospitalized in our section and because of the massive difficult workload our colleagues cannot devote that much time to each and every patient, and when sufficient time is not spent with patients, sufficient care cannot be provided as well (p 20).

The homogeneity of demographic and cultural characteristics, such as the closeness of beliefs and language, facilitates the process of providing palliative care by influencing building emotional connections, having a core value in care and reinforcing positive mindset. In this regard, a daughter of a patient stated:

When the nurse, physician or other staff members share the same language as ours here and we come from the same area, it is much easier to connect with them (p 17).

Also, the referrals between different groups of care providers and their cooperation improve the psycho‐emotional recovery as it positively impacts emotional connection. A RN stated:

When our patients encounter mental problems, we get help from our psychologist colleagues and they provide consults. These things all help the patients to feel that a group of professionals are taking care of them, which in turn contributes to building better connections (p 18).

### 4.7 Implications of strategies

Findings showed that the patients who had received palliative care by means of the strategies mentioned above, obtained a state of inner peace and this was realized through the development of the feelings of satisfaction, trust, sense of worth, hope and a sense of well‐being.

The doctor… gives a lot of hope. She treats me really well, and when she's like that I feel like I can trust her and feel a lot hopeful. I calm down and feel peaceful and don't think about negative stuff anymore (p 16).

In addition to this positive outcome, due to the constant presence of patients' family members and the pressure that caring for these patients imposes on their families, patients may feel like a burden on their families.

Because of my condition, my family members try to always be beside me and they are under pressure. They have put their own things aside and are just always here beside me and this kind of upsets me (p 9).

On the other hand, care providers who seek to establish an emotional connection with patients, sometimes undergo psychological distress themselves and are concerned about the aggravation of these distress in the continuation of the care of people with cancer.

Building a connection with such patients, especially when they are drowning in problems, and when the connection is going to be deep and for a longer period, it affects you and your mental state. For example, I once had this patient who was dealing with a lot of problems and I couldn't get her out of my mind even when I wanted to go to sleep; now, just imagine taking care of such a patient for a number of years. Seriously, what would it do to our mentality? (p 6).

### 4.8 Integrating categories

**FIGURE 1:**
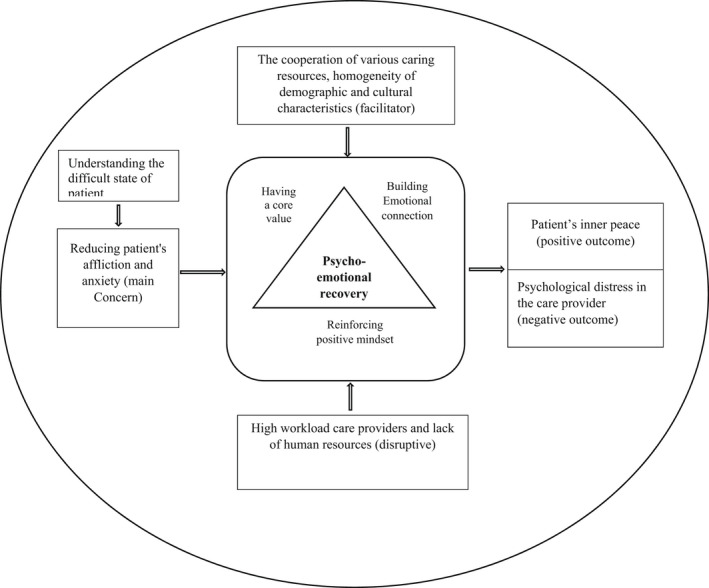
The relation between concepts in the underlying theory of Psycho‐emotional recovery.

Finally, in order to incorporate the findings and discuss the substantive theory, first the core category had to be determined as it played a key role in linking and incorporating the findings; then the underlying theory had to be explained. To find the core category, the memos have been reviewed and the main line of narration has been written. Based on the line of narration, the concepts explored and the relationship between concerns, strategies and consequences, psycho‐emotional recovery was considered to be a core category. All concepts related to different strategies, such as emotional connection, having a core value in care, and reinforcing positive mindset, alongside all the concerns and consequences explored, are interlinked at a higher level of psycho‐emotional recovery. This is due to the fact that all concepts related to the provision of palliative care have been placed next to each other because care providers have focused their services on the recovery of the emotional mental state of patients. Finally, on the basis of the role held by each concept in relation to the core category and the other concepts, the manner in which they are prioritized, also on the basis of relational propositions and the type of relationship that occurs between concepts and psycho‐emotional recovery, the categories have been related and incorporated on the basis of the core category. The substantive theory of the provision of palliative care in Iranian people with cancer was found after refining these categories, which is explained here under:

In the treatment of people with cancer, palliative care providers understand and observe the lengthy tedious nature of their disease and treatment, patients drown in negative emotions, the patient's non‐compliance because of their condition and patient's vague future and opportunity of death. In view of the above, the main concern of palliative care professionals is to “reducing the affliction and anxiety of patients” while delivering care and to apply the psycho‐emotional recovery approach. This strategy involves techniques such as building emotional connections, having a core value of care and reinforcing positive mindset. The homogeneity of demographic and cultural characteristics and the cooperation of various care resources have strengthened the psycho‐emotional recovery strategy, and factors such as the high workload of care providers and the lack of human resources have actually disrupted the psycho‐emotional recovery strategy. Providing palliative care with a focus on the psycho‐emotional recovery approach provides a sense of inner peace in patients. However, in addition to this positive outcome, patients may feel disappointed by the continued involvement of their families and feel a burden on their families due to the need for continuing care. In addition, palliative care providers will undergo psychological distress in the palliative care delivery process (Figure 1).

## 5 DISCUSSION

In the present study, the qualitative research approach of grounded theory was used to explore how to provide palliative care and the care process. The grounded theory is an appropriate research method for examining processes in social interaction; because palliative care is a process of interactions, behaviours, and practices that can be explored with a grounded theory approach. The present study represents the process of providing palliative care as patients' psycho‐emotional recovery. In line with the present study, Soanes and Gibson (2018) has interpreted the meaning of supportive care in people with cancer to be patient identity preservation by which care providers try to maintain patients' identities in a changed situation where the patient undergoes an exhausting condition. The changed situation in the above study indicates that patients have suffered under different conditions. Accordingly, the findings of the present study showed that care providers understand the difficult state of patient and their main concern is to reduce the patients' affliction. This main concern is one of the most statistically significant ethical aspects in the process of providing patient‐centred care. (Bélanger et al., 2016).

Nwozichi (2019) characterized the process of providing palliative care to people with cancer as the concept of knowing, revealing, and humanizing, which creates a positive meaning in a person's life. Knowing and revealing are two subcategories of reinforcing a positive mindset. Furthermore, the concept of humanizing is consistent with the concept of central value in the present study. Creating a positive meaning in patients' lives reflects on providing a psychological support for patients, which is mentioned in the above study. Rahnama et al. examined the process of providing care to people with cancer with a focus on spiritual rehabilitation. The results of their study showed that the care process mainly involved supporting patients, which was fulfilled by direct and indirect support for nurses. Indirect support for nurses empowers them in the process of caring and rehabilitating people with cancer (Rahnama et al., 2015). The result of our study was contrary to this specific finding of Rahnama et al. Nurses in the present study suffered from psychological distress, which indicates that they were not receiving sufficient support in the process, which can also be due to managerial factors such as lack of human resources. In addition, in order to support nurses, the results of various studies have referred to the supportive role of management and leadership (Ferris et al., 2018; Klarare et al., 2020).

The psycho‐emotional recovery of patients in the present study is done through three important strategies: building emotional connection, having a core value in care and reinforcing positive mindset. Seccareccia et al. (2015) found that the establishment of a close relationship with the patient gives patients a sense of belonging. It also makes them feel that they are provided with meaningful and real care. In line with the present study, a meta‐synthesis by Sekse et al. (2018) emphasized that spending more time with patients and maintaining a regular relationship with them helps nurses and patients to develop a special connection with each other. This special connection is expected to have the features that have been mentioned in the present study in the concept of emotional connection. It can be inferred that establishing a relationship between nurses and patients relies on the personal characteristics of individuals and the culture of the health care. When an emotional connection is developed between a patient and a care provider, it indicates that they have become closer on a more personal level, which has proven to be an effective method when trying to reduce patient's anxiety about their disease and care process. It has also helped care providers to deliver their service more effectively. In terms of palliative care in the present study, the relationship between care providers and patients has developed emotionally under the influence of the homogeneity of socio‐cultural characteristics. One of the important consequences following the establishment of emotional connection was psychological distress in care providers. Studies have shown that working and having a long‐term relationship with patients who are in a life‐threatening medical condition can cause emotional and psychological distress in care providers, which ultimately affects the process of effective care (Melvin, 2015; Slocum‐Gori et al., 2013; Whitebird et al., 2013). Valente and Teixeira (2009) showed that cancer caregivers experience negative emotions that affect their personal lives and further create a variety of psychological pressures. Therefore, it is necessary to use relevant strategies to overcome this issue. Orellana‐Rios et al. (2018) found that providing conscious empathy and relieving minds are the most salient strategies that can be used to overcome such stresses. In a systematic review, Hill et al. (2016) emphasized that about emotional and psychological distress in care providers, measures such as relaxation techniques, training, cognitive support, education and targeted stress develop psychological flexibility and reduce psychological distress.

Another strategy for psycho‐emotional recovery in the present study was setting a central value in care, which is characterized by spirituality and respect for different beliefs and dignified care. Other studies have also investigated the role of spirituality as being a factor that improves quality of life, cope with concerns and provide hope for patients (Bai & Lazenby, 2015; Ferrell et al., 2013; Fitch & Bartlett, 2019; Meireles et al., 2015; Puchalski, 2012). Despite the common intrinsic values of patients receiving palliative care, there are also some differences depending on the cultural and religious contexts (Herlianita et al., 2018; Lee, 2019; Martin & Barkley Jr, 2016). Patients' beliefs in different contexts affect their values; therefore, different strategies may be used in different areas. The findings of the present study showed that dignity‐based care is one of the important components of value‐based care. Maintaining human dignity as a moral value in palliative care is one of the important concepts in patient‐centred care process (Guo & Jacelon, 2014; Pringle et al., 2015). It can be inferred that the central value of care is human dignity, with emphasis on solidarity between patients and care providers and with the goal of rehabilitating patients emotionally. The solidarity and relationship with patients also involves mutual respect. This respectful relationship builds trust and support, which ultimately leads to patients' comfort.

Reinforcing positive mentality was one of the salient strategies in psycho‐emotional recovery as mentioned in the present study. This strategy involved inducing reality to the patient, providing care awareness, maintaining real hope and raising patient's spirit. Along with the concept of inducing reality and providing awareness, one of the psychological treatment techniques used for people with cancer is acceptance and commitment‐based therapy. Being prepared to experience unpleasant feelings and not avoiding them lead to psychological flexibility in patients (Clarke et al., 2012; Kahl et al., 2012). In other words, this treatment strategy is the process of reducing the patient's anxiety and psychological suffering. It also eliminates unwanted thoughts, emotions and unpleasant feelings, strengthens psychological flexibility and establishes the ability to change one's behaviour.

Reinforcing a positive mindset through giving real hope to patients helps them to cope with the pain of their illness and continue their treatment. It also prevents them from exhaustion and passivity. Soundy et al. (2014) found that in the process of providing care for long‐term illness, despair caused more problems such as pain, lower self‐esteem, anxiety and depression. Therefore, hope is a valuable insight that energizes patients. This energy is more concerned with the patient's psychological dimension. Although patients may find hope in denying the reality, when combined with concept of inducing reality, this form of hope in palliative care leads to the formation of true hope. Furthermore, raising morale, improving positive thinking and creating real hope in patients were said by the participants of the present study. Given that the exhausting process of cancer has harmful effects on patients' quality of life, raising the patient's morale and positive thinking protects them from these harmful effects and helps patients to overcome their suffering and achieve inner peace. Raising the morale of people with cancer is a part of positive psychology, in which an emphasis is equally placed on negative and positive experiences (Coyne & Tennen, 2010). Cancer has devastating effects with an inevitably exhaustive nature. However, palliative care providers focus on the positive consequences and experiences of patients to empower the patient. In other words, patients' achievement of inner peace or turmoil is influenced by focusing on the positive points and strengthening the morale, or through focusing on negative and exhausting experiences.

### 5.1 Limitation of the study

The limitation of the present study was the lack of sufficient participants, especially in the professions of social worker, religious counsellor and psychologist; because in these professions there was not enough manpower in the palliative care centres. In the present study, in order to help diversify sampling, only one person from each of the mentioned professions was interviewed.

## 6 CONCLUSION

The underlying theory of psycho‐emotional recovery provides a new perspective on why and how palliative care is provided in Iran. In Iranian palliative care context, care providers use building emotional connection, reinforcing patient's positive mindset, having a core value in care strategies to have effective care of patients and the core of these strategies is the psycho‐emotional recovery that is done to reduce the reduce the affection and anxiety of patients.

## 7 RELEVANCE TO CLINICAL PRACTICE

Psycho‐emotional recovery strategies can be used during the development and monitoring of palliative care in the care system. Given the fact that palliative care is a necessity in the management of people with cancer, the “psycho‐emotional recovery” theory can be used as a guide for describing and expanding nurses' roles in palliative care delivery and clinical guidelines, and planning educational programs for nursing students and staff nurses. The present study showed that care providers were less supported in their workplace and some suffered from psychological distress. Traumatization of care providers could impair the quantity and quality of care. Therefore, it is necessary to eliminate psychological trauma in care providers by recruiting more human resources into the program and also improving the work environment. Conducting action research studies in line with the present study can reveal the strengths and weaknesses of the present theory and facilitate its practical use in the context of care.

## CONFLICT OF INTEREST

The authors have no conflicts of interest to declare that are relevant to the content of this article.

## ETHICAL APPROVAL

Participants were assured that their statements were kept confidential; So that their data has been managed, analysed, and reported anonymously, using numbers.

## Data Availability

The data that support the findings of this study are available from the corresponding author upon reasonable request

## References

[nop21357-bib-0001] Abu Sharour, L. , Malak, M. , Subih, M. , & Bani Salameh, A. (2020). Quality of life, care needs, and information needs among patients diagnosed with cancer during their treatment phase. Psychology, Health & Medicine, 25(2), 252–258. 10.1080/13548506.2019.1699660 31795738

[nop21357-bib-0002] Agarwal, R. , & Epstein, A. S. (2018). Advance care planning and end‐of‐life decision making for patients with cancer. Seminars in Oncology Nursing, 34(3), 316–326. 10.1016/j.soncn.2018.06.012 30100366 PMC6156999

[nop21357-bib-0003] Bai, M. , & Lazenby, M. (2015). A systematic review of associations between spiritual well‐being and quality of life at the scale and factor levels in studies among patients with cancer. Journal of Palliative Medicine, 18(3), 286–298. 25303461 10.1089/jpm.2014.0189 PMC4348086

[nop21357-bib-0004] Balducci, L. , & Dolan, D. (2016). Palliative care of cancer in the older patient. Current Oncology Reports, 18(12), 70. 10.1007/s11912-016-0557-2 27812859

[nop21357-bib-0005] Bélanger, E. , Rodríguez, C. , Groleau, D. , Légaré, F. , Macdonald, M. E. , & Marchand, R. (2016). Patient participation in palliative care decisions: an ethnographic discourse analysis. International Journal of Qualitative Studies on Health and Well‐Being, 11(1), 32438. 27882864 10.3402/qhw.v11.32438 PMC5122231

[nop21357-bib-0006] Boeije, H. (2009). Analysis in qualitative research. Sage publications.

[nop21357-bib-0007] Chamberlain‐Salaun, J. , Mills, J. , & Usher, K. (2013). Linking symbolic interactionism and grounded theory methods in a research design: from Corbin and Strauss' assumptions to action. SAGE Open, 3(3), 2158244013505757.

[nop21357-bib-0008] Corbin, J. M. , & Strauss, A. L. (2015). Basics of qualitative research: Techniques and procedures for developing grounded theory, 4th ed. (pp. 1–431). Los Angeles (CA): Sage.

[nop21357-bib-0009] Clarke, S. , Kingston, J. , Wilson, K. G. , Bolderston, H. , & Remington, B. (2012). Acceptance and commitment therapy for a heterogeneous group of treatment‐resistant clients: A treatment development study. Cognitive and Behavioral Practice, 19(4), 560–572.

[nop21357-bib-0010] Coyne, J. C. , & Tennen, H. (2010). Positive psychology in cancer care: Bad science, exaggerated claims, and unproven medicine. Annals of Behavioral Medicine, 39(1), 16–26. 20146038 10.1007/s12160-009-9154-z PMC2858800

[nop21357-bib-0011] Dahlin, C. (2015). Palliative care: Delivering comprehensive oncology nursing care. Seminars in Oncology Nursing, 31, 327–337. 26525732 10.1016/j.soncn.2015.08.008

[nop21357-bib-0012] Deitrick, L. M. , Rockwell, E. H. , Gratz, N. , Davidson, C. , Lukas, L. , Stevens, D. , Fitzgerald, G. , Naugle, M. , Wolf, J. , & Sikora, B. (2011). Delivering specialized palliative care in the community: A new role for nurse practitioners. Advances in Nursing Science, 34(4), E23–E36. 22067236 10.1097/ANS.0b013e318235834f

[nop21357-bib-0013] Ferrell, B. , Otis‐Green, S. , & Economou, D. (2013). Spirituality in cancer care at the end of life. The Cancer Journal, 19(5), 431–437. 24051617 10.1097/PPO.0b013e3182a5baa5

[nop21357-bib-0014] Ferris, F. D. , Moore, S. Y. , Callaway, M. V. , & Foley, K. M. (2018). Leadership development initiative: Growing global leaders… advancing palliative care. Journal of Pain and Symptom Management, 55(2), S146–S156. 28803073 10.1016/j.jpainsymman.2017.05.011

[nop21357-bib-0015] Fitch, M. I. , & Bartlett, R. (2019). Patient perspectives about spirituality and spiritual care. Asia‐Pacific Journal of Oncology Nursing, 6(2), 111–121. 30931354 10.4103/apjon.apjon_62_18 PMC6371668

[nop21357-bib-0016] Guo, Q. , & Jacelon, C. S. (2014). An integrative review of dignity in end‐of‐life care. Palliative Medicine, 28(7), 931–940. 24685648 10.1177/0269216314528399

[nop21357-bib-0017] Herlianita, R. , Yen, M. , Chen, C.‐H. , Fetzer, S. J. , & Lin, E. C.‐L. (2018). Perception of spirituality and spiritual care among Muslim nurses in Indonesia. Journal of Religion and Health, 57(2), 762–773. 28647910 10.1007/s10943-017-0437-6

[nop21357-bib-0018] Hill, R. C. , Dempster, M. , Donnelly, M. , & McCorry, N. K. (2016). Improving the wellbeing of staff who work in palliative care settings: A systematic review of psychosocial interventions. Palliative Medicine, 30(9), 825–833. 26944534 10.1177/0269216316637237

[nop21357-bib-0019] Imhof, L. , Kipfer, S. , & Waldboth, V. (2016). Nurse‐led palliative care services facilitate an interdisciplinary network of care. International Journal of Palliative Nursing, 22(8), 404–410. 27568780 10.12968/ijpn.2016.22.8.404

[nop21357-bib-0020] Iskandar, A. C. , Rochmawati, E. , & Wiechula, R. (2020). Patient's experiences of suffering across the cancer trajectory: A qualitative systematic review protocol. Journal of Advanced Nursing\, 77, 1037–1042. 10.1111/jan.14628 33210384

[nop21357-bib-0021] Jacobsen, P. B. , DeRosa, A. P. , Henderson, T. O. , Mayer, D. K. , Moskowitz, C. S. , Paskett, E. D. , & Rowland, J. H. (2018). Systematic review of the impact of cancer survivorship care plans on health outcomes and health care delivery. Journal of Clinical Oncology, 36(20), 2088–2100. 29775389 10.1200/JCO.2018.77.7482 PMC6036622

[nop21357-bib-0022] Kahl, K. G. , Winter, L. , & Schweiger, U. (2012). The third wave of cognitive behavioural therapies: What is new and what is effective? Current Opinion in Psychiatry, 25(6), 522–528. 22992547 10.1097/YCO.0b013e328358e531

[nop21357-bib-0023] Khoshnazar, T. A. K. , Rassouli, M. , Akbari, M. E. , Lotfi‐Kashani, F. , Momenzadeh, S. , Haghighat, S. , & Sajjadi, M. (2016). Structural challenges of providing palliative care for patients with breast cancer. Indian Journal of Palliative Care, 22(4), 459. 27803569 10.4103/0973-1075.191828 PMC5072239

[nop21357-bib-0024] Klarare, A. , Lind, S. , Hansson, J. , Fossum, B. , Fürst, C. J. , & Lundh Hagelin, C. (2020). Leadership in specialist palliative home care teams: A qualitative study. Journal of Nursing Management, 28(1), 102–111. 31868284 10.1111/jonm.12902

[nop21357-bib-0025] Lee, Y.‐H. (2019). Spiritual care for cancer patients. Asia‐Pacific Journal of Oncology Nursing, 6(2), 101–103. 30931352 10.4103/apjon.apjon_65_18 PMC6371666

[nop21357-bib-0026] Levit, L. A. , Balogh, E. , Nass, S. J. , & Ganz, P. (2013). Delivering high‐quality cancer care: charting a new course for a system in crisis. National Academies Press. 24872984

[nop21357-bib-0027] Martin, E. M. , & Barkley, T. W., Jr. (2016). Improving cultural competence in end‐of‐life pain management. Nursing, 46(1), 32–41. 10.1097/01.NURSE.0000475480.75266.9a 26626723

[nop21357-bib-0028] Meireles, C. B. , Maia, L. C. , Miná, V. A. L. , Novais, M. D. S. M. C. , Peixoto, J. A. C. , Cartaxo, M. A. B. S. , Ferreira de Lima, J. M. , Vieria dos Santos, F. A. , de Matos Cassiano, C. J. , Pinheiro, P. G. , & Neto, M. L. R. (2015). Influence of spirituality in pediatric cancer management: A systematic review. International Archives of Medicine, 8, 1–13.

[nop21357-bib-0029] Melvin, C. S. (2015). Historical review in understanding burnout, professional compassion fatigue, and secondary traumatic stress disorder from a hospice and palliative nursing perspective. Journal of Hospice & Palliative Nursing, 17(1), 66–72.

[nop21357-bib-0030] Mojen, L. K. (2017). Palliative care in Iran: The past, the present and the future. Supportive & Palliative Care in Cancer, 1(1), 8–11.

[nop21357-bib-0031] Nwozichi, C. U. (2019). Toward a germinal theory of knowing‐revealing‐humanizing as expressions of caring in cancer palliative care. Asia‐Pacific Journal of Oncology Nursing, 6(3), 269–276. 31259223 10.4103/apjon.apjon_9_19 PMC6518987

[nop21357-bib-0032] Orellana‐Rios, C. L. , Radbruch, L. , Kern, M. , Regel, Y. U. , Anton, A. , Sinclair, S. , & Schmidt, S. (2018). Mindfulness and compassion‐oriented practices at work reduce distress and enhance self‐care of palliative care teams: a mixed‐method evaluation of an “on the job” program. BMC Palliative Care, 17(1), 3. 10.1186/s12904-017-0219-7 PMC5501358 28683799

[nop21357-bib-0033] Partridge, A. H. , Seah, D. S. , King, T. , Leighl, N. B. , Hauke, R. , Wollins, D. S. , & Von Roenn, J. H. (2014). Developing a service model that integrates palliative care throughout cancer care: the time is now. Journal of Clinical Oncology, 32(29), 3330–3336. 25199756 10.1200/JCO.2013.54.8149

[nop21357-bib-0034] Polit, D. F. , & Beck, C. T. (2008). Nursing research: Generating and assessing evidence for nursing practice. Lippincott Williams & Wilkins.

[nop21357-bib-0035] Pringle, J. , Johnston, B. , & Buchanan, D. (2015). Dignity and patient‐centred care for people with palliative care needs in the acute hospital setting: A systematic review. Palliative Medicine, 29(8), 675–694. 25802322 10.1177/0269216315575681

[nop21357-bib-0036] Puchalski, C. M. (2012). Spirituality in the cancer trajectory. Annals of Oncology, 23(suppl_3), 49–55. 22628416 10.1093/annonc/mds088

[nop21357-bib-0037] Rahnama, M. , Fallahi, K. M. , Seyed, B. M. S. , & Ahmadi, F. (2014). Designing a model for spiritual care in rehabilitation of cancer patients. Medical ‐ Surgical Nursing Journal, 3(2), 61–70.

[nop21357-bib-0038] Rahnama, M. , Fallahi, K. M. , Seyed, B. M. S. , & Ahmadi, F. (2015). The process of spiritual care in rehabilitation of cancer patients: A grounded theory study. Medical ‐ Surgical Nursing Journal, 4(3), 1–12.

[nop21357-bib-0039] Rassouli, M. , & Sajjadi, M. (2016). Palliative care in Iran: Moving toward the development of palliative care for cancer. American Journal of Hospice and Palliative Medicine, 33(3), 240–244. 25492970 10.1177/1049909114561856

[nop21357-bib-0040] Romero, Y. , Trapani, D. , Johnson, S. , Tittenbrun, Z. , Given, L. , Hohman, K. , Stevens, L. , Torode, J. S. , Boniol, M. , & Ilbawi, A. M. (2018). National cancer control plans: a global analysis. The Lancet Oncology, 19(10), e546–e555. 10.1016/S1470-2045(18)30681-8 30268693

[nop21357-bib-0041] Salins, N. , Ramanjulu, R. , Patra, L. , Deodhar, J. , & Muckaden, M. A. (2016). Integration of early specialist palliative care in cancer care and patient related outcomes: A critical review of evidence. Indian Journal of Palliative Care, 22(3), 252. 27559252 10.4103/0973-1075.185028 PMC4973484

[nop21357-bib-0042] Seccareccia, D. , Wentlandt, K. , Kevork, N. , Workentin, K. , Blacker, S. , Gagliese, L. , Grossman, D. , & Zimmermann, C. (2015). Communication and quality of care on palliative care units: A qualitative study. Journal of Palliative Medicine, 18(9), 758–764. 26069934 10.1089/jpm.2014.0408

[nop21357-bib-0043] Sekse, R. J. T. , Hunskår, I. , & Ellingsen, S. (2018). The nurse's role in palliative care: A qualitative meta‐synthesis. Journal of Clinical Nursing, 27(1–2), e21–e38. 28695651 10.1111/jocn.13912

[nop21357-bib-0044] Seyedfatemi, N. , Borimnejad, L. , Hamooleh, M. M. , & Tahmasebi, M. (2014). Iranian nurses' perceptions of palliative care for patients with cancer pain. International Journal of Palliative Nursing, 20(2), 69–74. 24577212 10.12968/ijpn.2014.20.2.69

[nop21357-bib-0045] Slocum‐Gori, S. , Hemsworth, D. , Chan, W. W. , Carson, A. , & Kazanjian, A. (2013). Understanding compassion satisfaction, compassion fatigue and burnout: A survey of the hospice palliative care workforce. Palliative Medicine, 27(2), 172–178. 22179596 10.1177/0269216311431311

[nop21357-bib-0046] Soanes, L. , & Gibson, F. (2018). Protecting an adult identity: A grounded theory of supportive care for young adults recently diagnosed with cancer. International Journal of Nursing Studies, 81, 40–48. 10.1016/j.ijnurstu.2018.01.010 29455009

[nop21357-bib-0047] Soundy, A. , Liles, C. , Stubbs, B. , & Roskell, C. (2014). Identifying a framework for hope in order to establish the importance of generalised hopes for individuals who have suffered a stroke. Advances in Medicine, 2014, 2014–2018. 10.1155/2014/471874 PMC4590961 26556412

[nop21357-bib-0048] Stjernswärd, J. , Foley, K. M. , & Ferris, F. D. (2007). The public health strategy for palliative care. Journal of Pain and Symptom Management, 33(5), 486–493. 17482035 10.1016/j.jpainsymman.2007.02.016

[nop21357-bib-0049] Valente, S. H. , & Teixeira, M. B. (2009). Phenomenological study about the nurse's home care for families of terminally ill patients. Revista da Escola de Enfermagem da USP, 43(3), 655–661. 10.1590/s0080-62342009000300022 19842599

[nop21357-bib-0050] Vinckx, M.‐A. , Bossuyt, I. , & Dierckx de Casterlé, B. (2018). Understanding the complexity of working under time pressure in oncology nursing: A grounded theory study. International Journal of Nursing Studies, 87, 60–68. 10.1016/j.ijnurstu.2018.07.010 30055374

[nop21357-bib-0051] Wang, T. , Molassiotis, A. , Chung, B. P. M. , & Tan, J.‐Y. (2018). Unmet care needs of advanced cancer patients and their informal caregivers: A systematic review. BMC Palliative Care, 17(1), 96. 10.1186/s12904-018-0346-9 30037346 PMC6057056

[nop21357-bib-0052] Whitebird, R. R. , Asche, S. E. , Thompson, G. L. , Rossom, R. , & Heinrich, R. (2013). Stress, burnout, compassion fatigue, and mental health in hospice workers in Minnesota. Journal of Palliative Medicine, 16(12), 1534–1539. 24199789 10.1089/jpm.2013.0202

